# Supplementary material for: The Global Research Status and Trends in Ice and Snow Sports Injuries from 1995 to 2022: A Bibliometric and Visualized Analysis
Source: Int J Environ Res Public Health. 2023 Feb 7;20(4):2880. doi: 10.3390/ijerph20042880 (PMC9957478; doi:10.3390/ijerph20042880)
Supplement: Supplementary file 1 [file ijerph-20-02880-s001.zip › ijerph-2085088-supplementary.pdf]

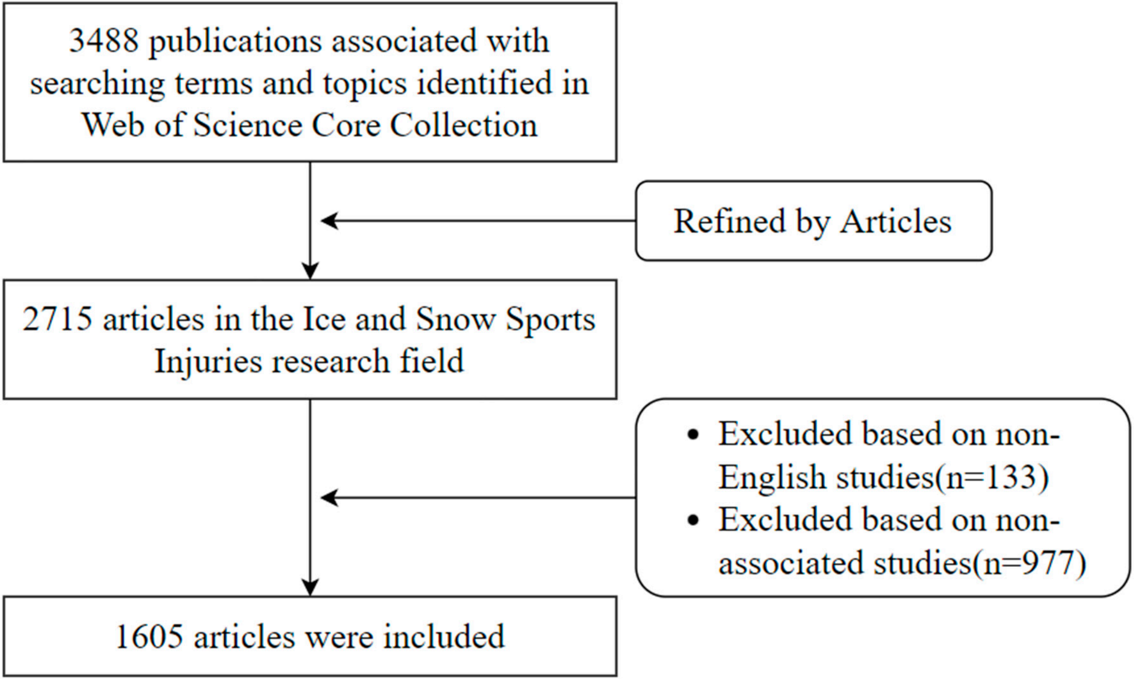
3488 publications associated with searching terms and topics identified in Web of Science Core Collection
Refined by Articles
2715 articles in the Ice and Snow Sports Injuries research field
Excluded based on non-English studies(n=133)
Excluded based on non-associated studies(n=977)
1605 articles were included
